# Supplementary material for: The climate opportunities and risks of contrail avoidance
Source: Nat Commun. 2026 Jan 28;17:2092. doi: 10.1038/s41467-026-68784-8 (PMC12953640; doi:10.1038/s41467-026-68784-8)
Supplement: Supplementary file 1 — Supplementary Information [file 41467_2026_68784_MOESM1_ESM.pdf]

The Climate Opportunities and Risks of Contrail Avoidance – Supplementary Information

Jessie R. Smith*[,1], Carla Grobler[2], Paul J. Hodgson[1], Jayant Mukhopadhaya[3], Marc Shapiro[4], Matteo Mirolo[4,5], Marc Stettler[6], Sebastian D. Eastham[7], Steven Barrett[1]

*	Corresponding author's email address: jrs201@cam.ac.uk.
1	Department of Engineering, University of Cambridge, Trumpington Street, Cambridge, CB2 1PZ, United Kingdom.
2	Department of Mechanical and Aeronautical Engineering, University of Pretoria, Hatfield, Pretoria, South Africa
3	International Council on Clean Transportation, 1500 K Street NW, Suite 650 Washington, DC 20005, United States.
4	Breakthrough Energy, Kirkland, Washington, United States.
5	Cambridge Institute for Sustainability Leadership, The Entopia Building 1 Regent Street, Cambridge, CB2 1GG, United Kingdom
6	Department of Civil and Environmental Engineering, Imperial College London, London, SW7 2AZ, United Kingdom.
7	Brahmal Vasudevan Institute for Sustainable Aviation, Department of Aeronautics, Imperial College London, London, SW7 2AZ, United Kingdom.

Supplementary Figure 1

Supplementary Figure 1 I: The global surface temperature rise due to contrails for various future scenarios related to flown distance and contrail forcing, where no contrail avoidance action is taken.

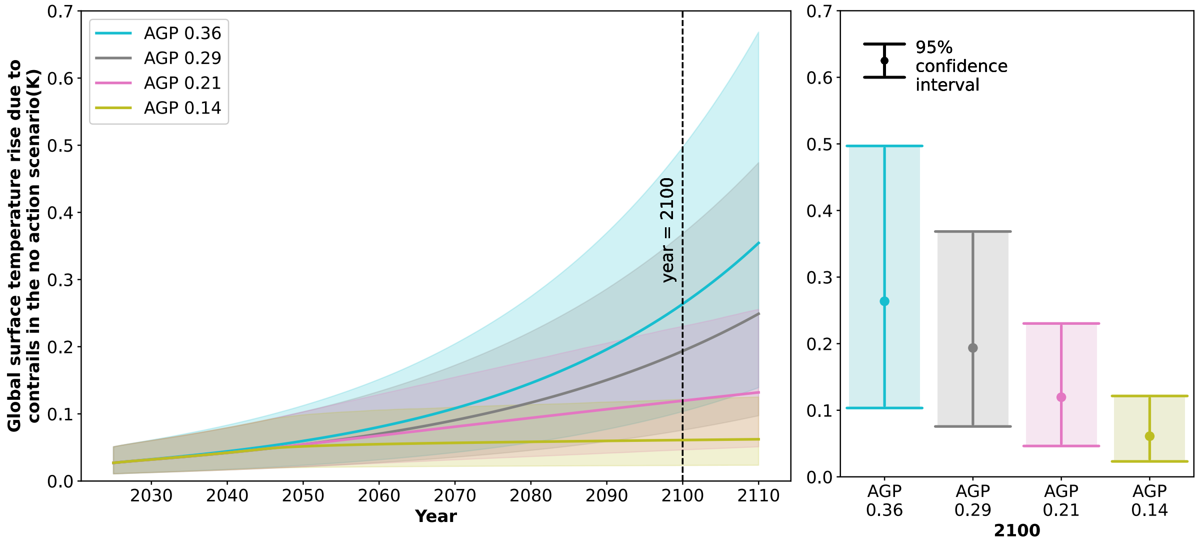

The distribution is presented from 2025 to 2110 (left) and for 2100 (right). The mean response (solid line) and 95% confidence intervals (shaded area) are depicted for various future aviation growth projections that relate to flown distance and contrail forcing. These projections are as follows: exponential growth in flown distance after 2045 where saturation in future contrail forcing effects is not assumed (AGP 0.36) and is assumed (AGP 0.29); and linear growth in flown distance after 2045 (AGP 0.21) and no growth in flown distance after 2045 (AGP 0.14), where saturation in future contrail forcing effects is assumed.

Supplementary Figure 1 II: The global surface temperature rise due to carbon dioxide for various future scenarios related to flown distance and contrail forcing, where no contrail avoidance action is taken.

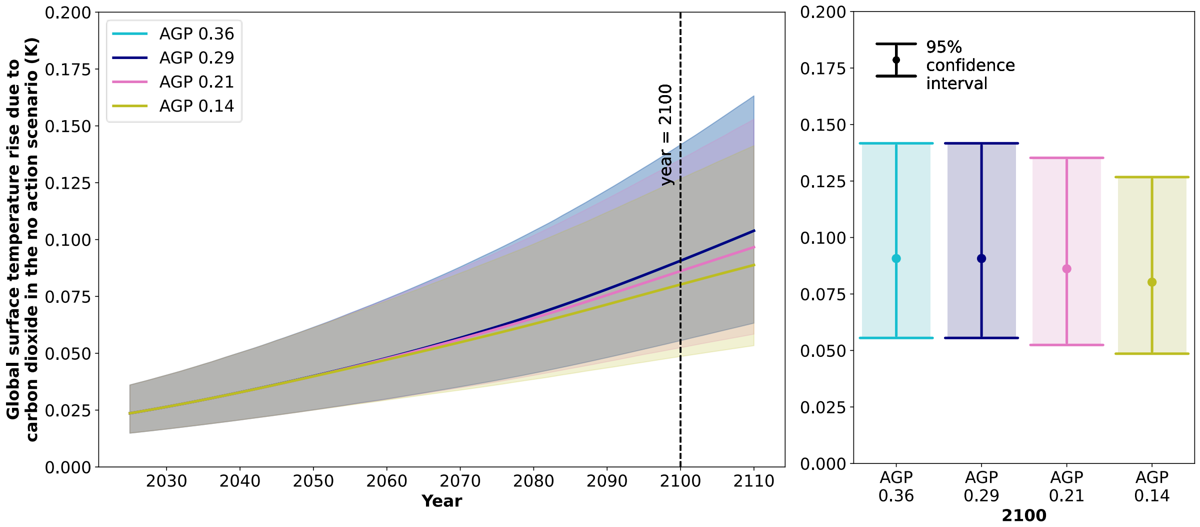

The distribution is presented from 2025 to 2110 (left) and for 2100 (right). The mean response (solid line) and 95% confidence intervals (shaded area) are depicted for various future aviation growth projections that relate to flown distance and contrail forcing. These projections are as follows: exponential growth in flown distance after 2045 where saturation in future contrail forcing effects is not assumed (AGP 0.36) and is assumed (AGP 0.29); and linear growth in flown distance after 2045 (AGP 0.21) and no growth in flown distance after 2045 (AGP 0.14), where saturation in future contrail forcing effects is assumed.

Supplementary Figure 2

Supplementary Figure 2 I: The global surface temperature rise in the scenario where no contrail avoidance action is taken.

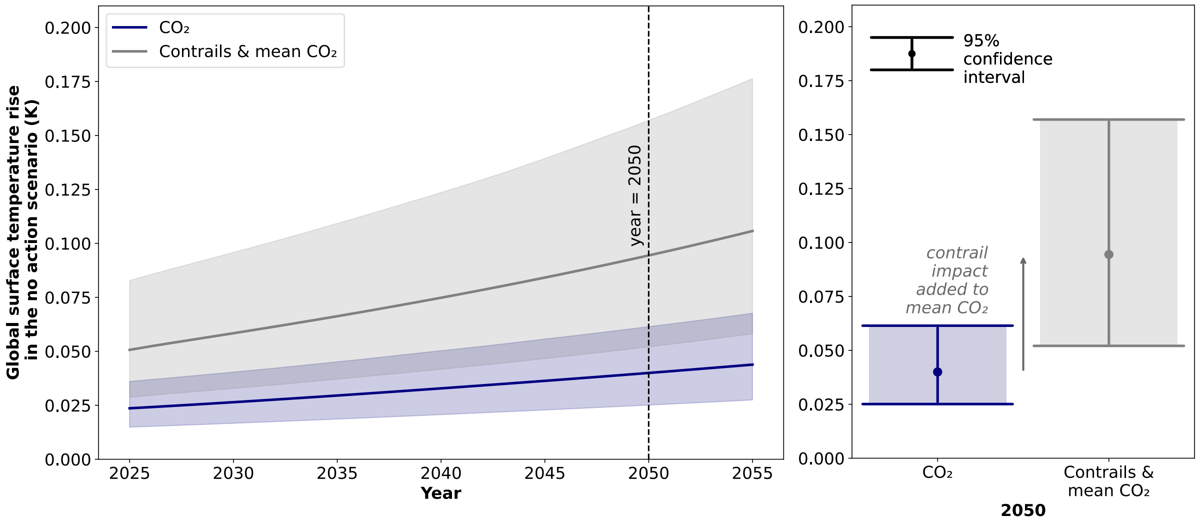

The distribution is presented from 2025 to 2055 (left) and for 2050 (right). The mean response (solid line) and 95% confidence intervals (shaded area) for contrails are depicted as an addition to the mean response for aviation CO_2 emissions.

The aviation growth projection (AGP) AGP 0.29 is assumed, i.e. exponential growth in flown distance beyond 2045 and a saturation in future contrail forcing effects.

Supplementary Figure 2 II: The global surface temperature rise in the scenario where no contrail avoidance action is taken.

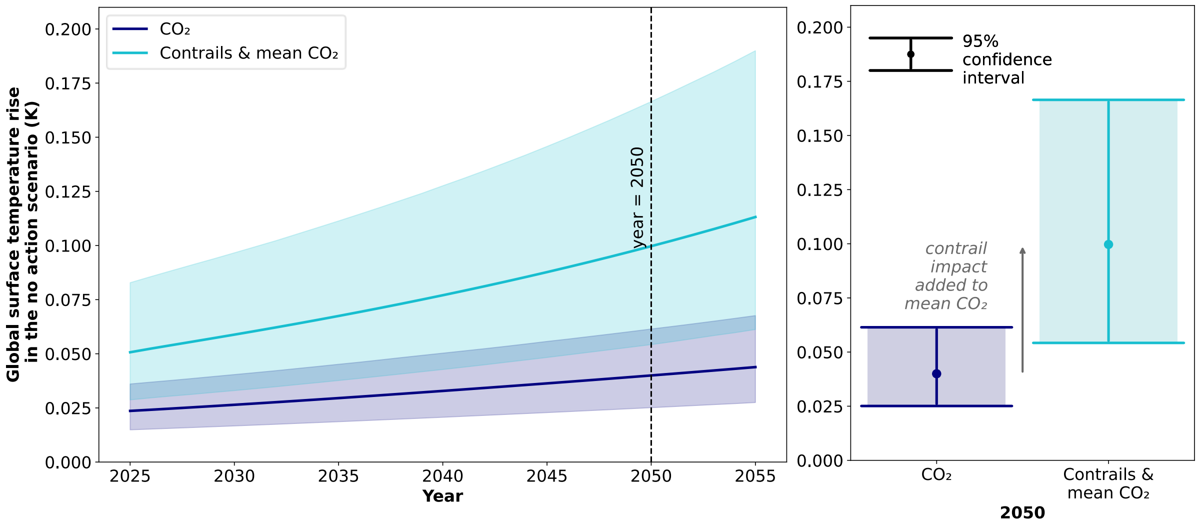

The distribution is presented from 2025 to 2055 (left) and for 2050 (right). The mean response (solid line) and 95% confidence intervals (shaded area) for contrails are depicted as an addition to the mean response for aviation CO_2 emissions.

The aviation growth projection (AGP) AGP 0.36 is assumed, i.e. exponential growth in flown distance beyond 2045 and no saturation in future contrail forcing effects.

Supplementary Figure 2 III: The global surface temperature rise in the scenario where no contrail avoidance action is taken.

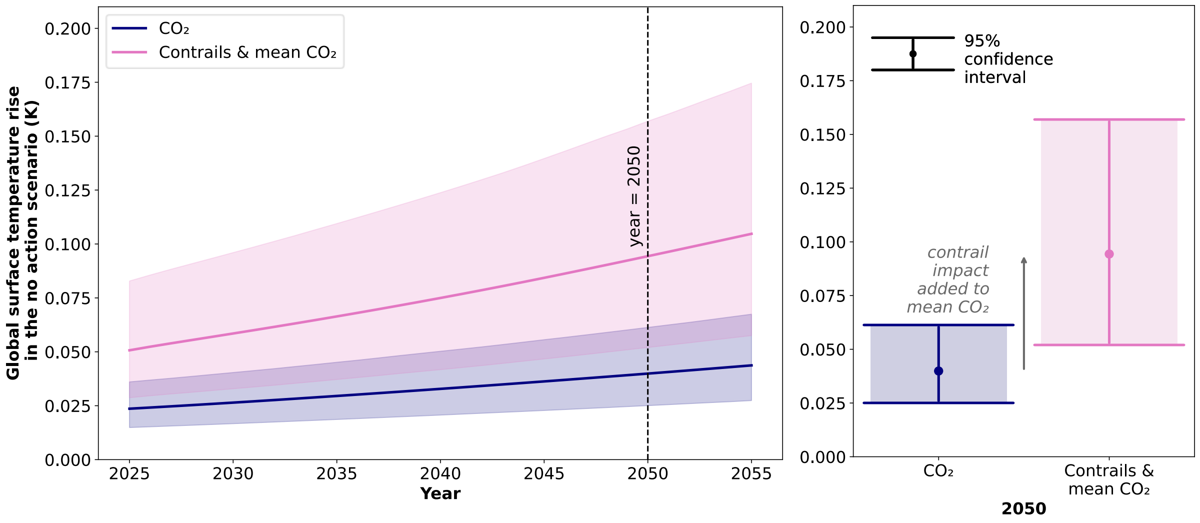

The distribution is presented from 2025 to 2055 (left) and for 2050 (right). The mean response (solid line) and 95% confidence intervals (shaded area) for contrails are depicted as an addition to the mean response for aviation CO_2 emissions.

The aviation growth projection (AGP) AGP 0.21 is assumed, i.e. linear growth in flown distance beyond 2045 and a saturation in future contrail forcing effects.

Supplementary Figure 2 IV: The global surface temperature rise in the scenario where no contrail avoidance action is taken.

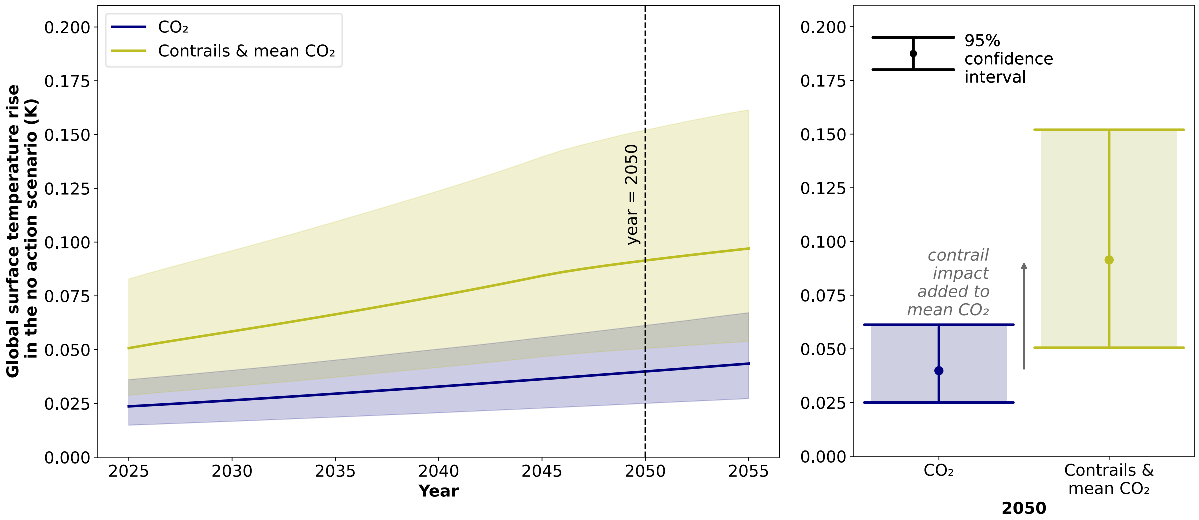

The distribution is presented from 2025 to 2055 (left) and for 2050 (right). The mean response (solid line) and 95% confidence intervals (shaded area) for contrails are depicted as an addition to the mean response for aviation CO_2 emissions.

The aviation growth projection (AGP) AGP 0.14 is assumed, i.e. no growth in flown distance beyond 2045 and a saturation in future contrail forcing effects.

Supplementary Figure 3

Supplementary Figure 3 I: The global surface temperature rise due to contrails, for contrail avoidance scenarios with different levels of effectiveness.

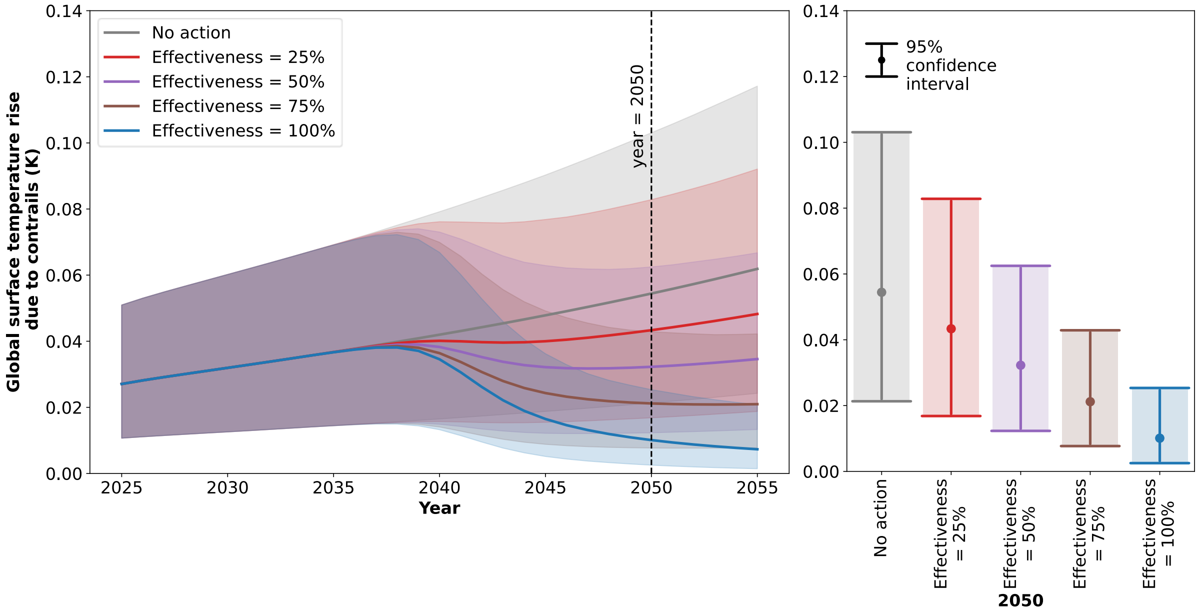

The distribution is presented from 2025 to 2055 (left) and for 2050 (right). The mean response (solid line) and 95% confidence intervals (shaded area) are depicted for contrail avoidance scenarios with levels of contrail avoidance effectiveness of 0% (here equivalent to no contrail avoidance action, i.e. “No action”), 25%, 50%, 75% and 100%. All scenarios have a 2035 start date, and progress from no adoption to fleet wide adoption over the course of 10 years.

The aviation growth projection (AGP) AGP 0.29 is assumed, i.e. exponential growth in flown distance beyond 2045 and a saturation in future contrail forcing effects.

Supplementary Figure 3 II: The global surface temperature rise due to contrails, for contrail avoidance scenarios with different levels of effectiveness.

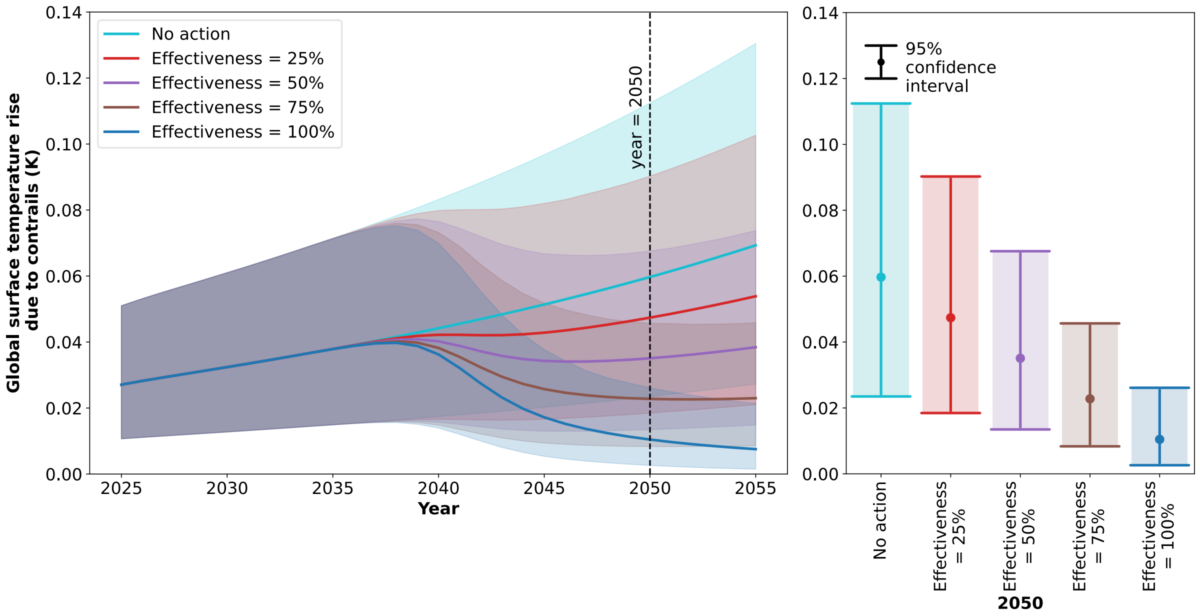

The distribution is presented from 2025 to 2055 (left) and for 2050 (right). The mean response (solid line) and 95% confidence intervals (shaded area) are depicted for contrail avoidance scenarios with levels of contrail avoidance effectiveness of 0% (here equivalent to no contrail avoidance action, i.e. “No action”), 25%, 50%, 75% and 100%. All scenarios have a 2035 start date, and progress from no adoption to fleet wide adoption over the course of 10 years.

The aviation growth projection (AGP) AGP 0.36 is assumed, i.e. exponential growth in flown distance beyond 2045 and no saturation in future contrail forcing effects.

Supplementary Figure 3 III: The global surface temperature rise due to contrails, for contrail avoidance scenarios with different levels of effectiveness.

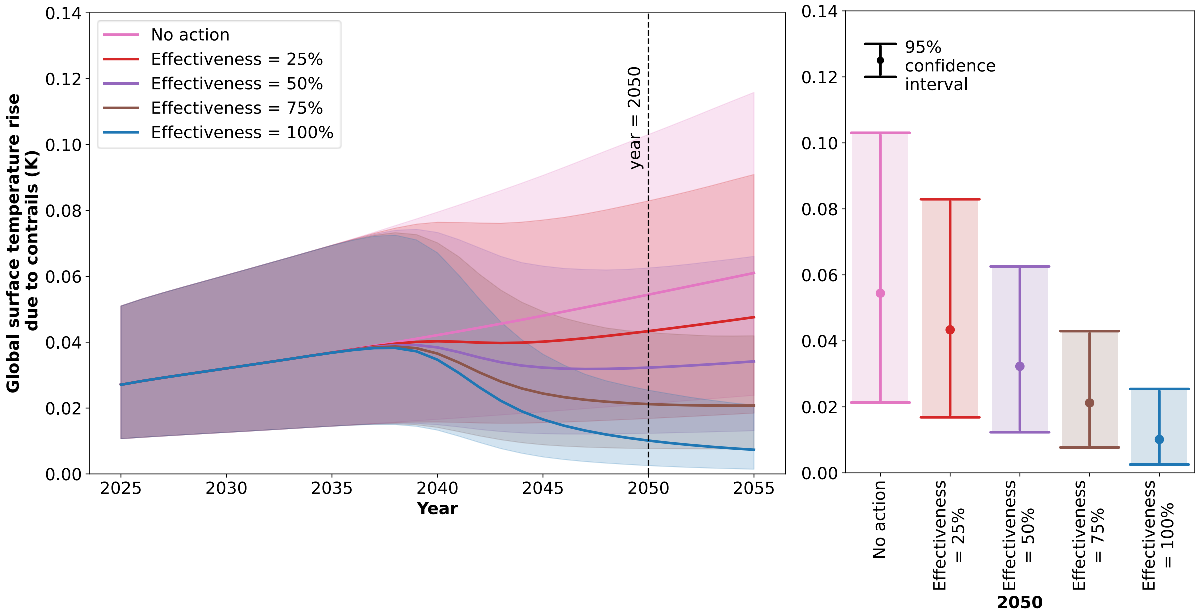

The distribution is presented from 2025 to 2055 (left) and for 2050 (right). The mean response (solid line) and 95% confidence intervals (shaded area) are depicted for contrail avoidance scenarios with levels of contrail avoidance effectiveness of 0% (here equivalent to no contrail avoidance action, i.e. “No action”), 25%, 50%, 75% and 100%. All scenarios have a 2035 start date, and progress from no adoption to fleet wide adoption over the course of 10 years.

The aviation growth projection (AGP) AGP 0.21 is assumed, i.e. linear growth in flown distance beyond 2045 and a saturation in future contrail forcing effects.

Supplementary Figure 3 IV: The global surface temperature rise due to contrails, for contrail avoidance scenarios with different levels of effectiveness.

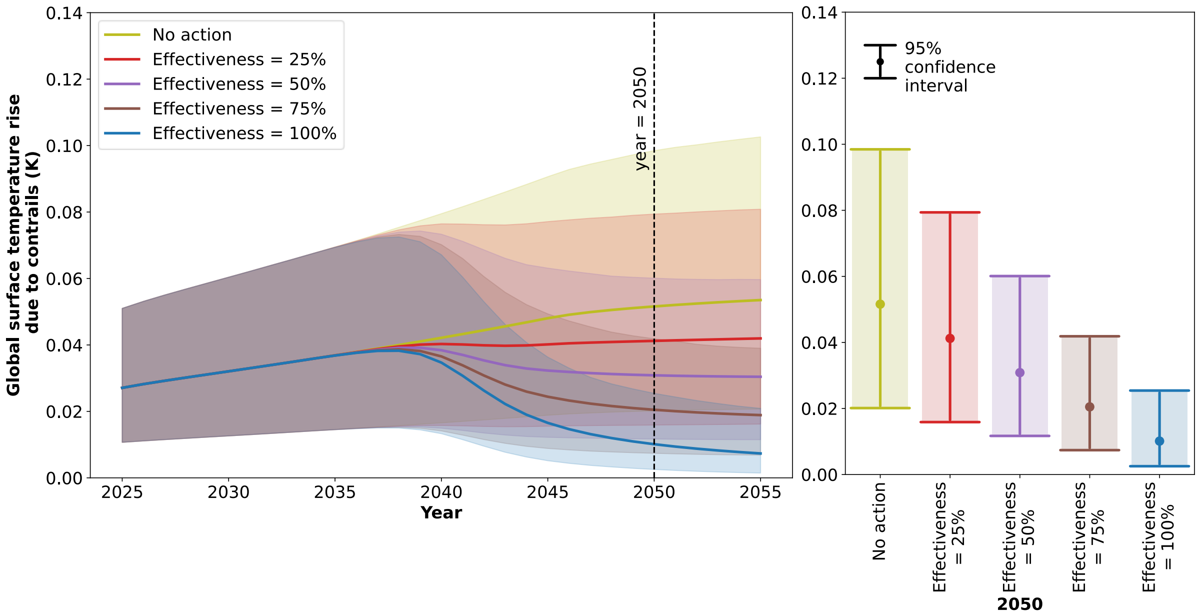

The distribution is presented from 2025 to 2055 (left) and for 2050 (right). The mean response (solid line) and 95% confidence intervals (shaded area) are depicted for contrail avoidance scenarios with levels of contrail avoidance effectiveness of 0% (here equivalent to no contrail avoidance action, i.e. “No action”), 25%, 50%, 75% and 100%. All scenarios have a 2035 start date, and progress from no adoption to fleet wide adoption over the course of 10 years.

The aviation growth projection (AGP) AGP 0.14 is assumed, i.e. no growth in flown distance beyond 2045 and a saturation in future contrail forcing effects.

Supplementary Figure 4

Supplementary Figure 4 I: The global surface temperature rise due to contrails, for contrail avoidance scenarios with different start dates.

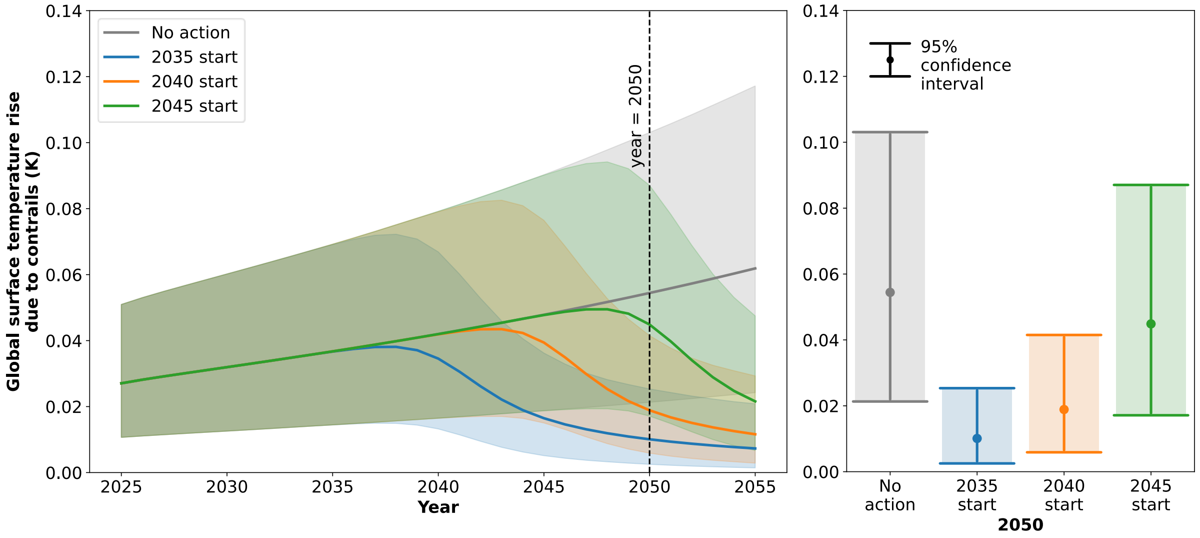

The distribution is presented from 2025 to 2055 (left) and for 2050 (right). The mean response (solid line) and 95% confidence intervals (shaded area) are depicted for contrail avoidance scenarios with start times of 2035, 2040, and 2045. All scenarios have 100% contrail avoidance effectiveness, and progress from no adoption to fleet wide adoption over the course of 10 years. The scenario where no contrail avoidance action is taken (No action) is also presented.

The aviation growth projection (AGP) AGP 0.29 is assumed, i.e. exponential growth in flown distance beyond 2045 and a saturation in future contrail forcing effects.

Supplementary Figure 4 II: The global surface temperature rise due to contrails, for contrail avoidance scenarios with different start dates.

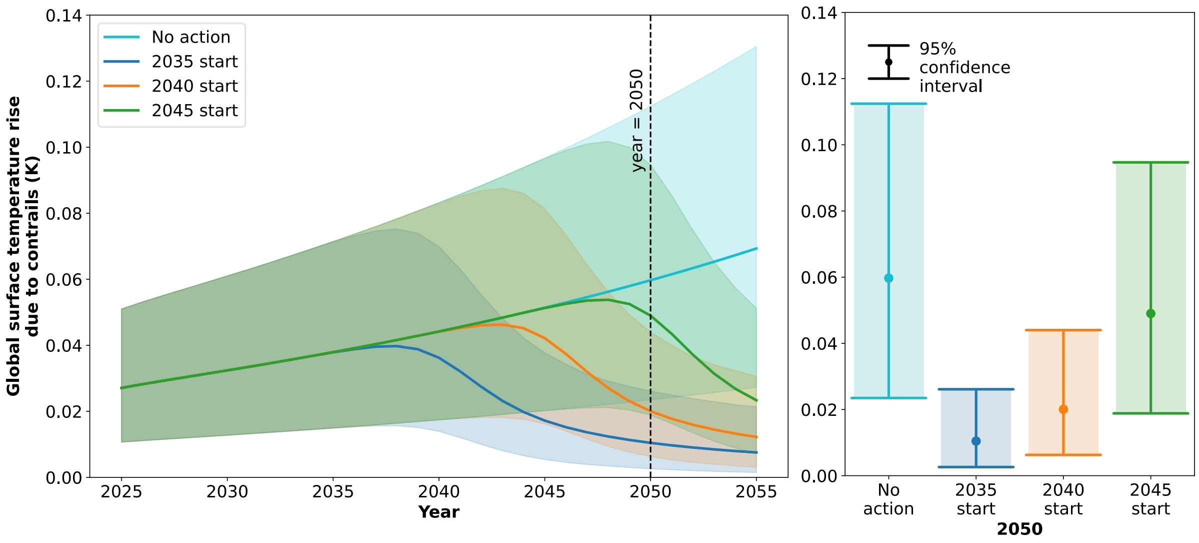

The distribution is presented from 2025 to 2055 (left) and for 2050 (right). The mean response (solid line) and 95% confidence intervals (shaded area) are depicted for contrail avoidance scenarios with start times of 2035, 2040, and 2045. All scenarios have 100% contrail avoidance effectiveness, and progress from no adoption to fleet wide adoption over the course of 10 years. The scenario where no contrail avoidance action is taken (No action) is also presented.

The aviation growth projection (AGP) AGP 0.36 is assumed, i.e. exponential growth in flown distance beyond 2045 and no saturation in future contrail forcing effects.

Supplementary Figure 4 III: The global surface temperature rise due to contrails, for contrail avoidance scenarios with different start dates.

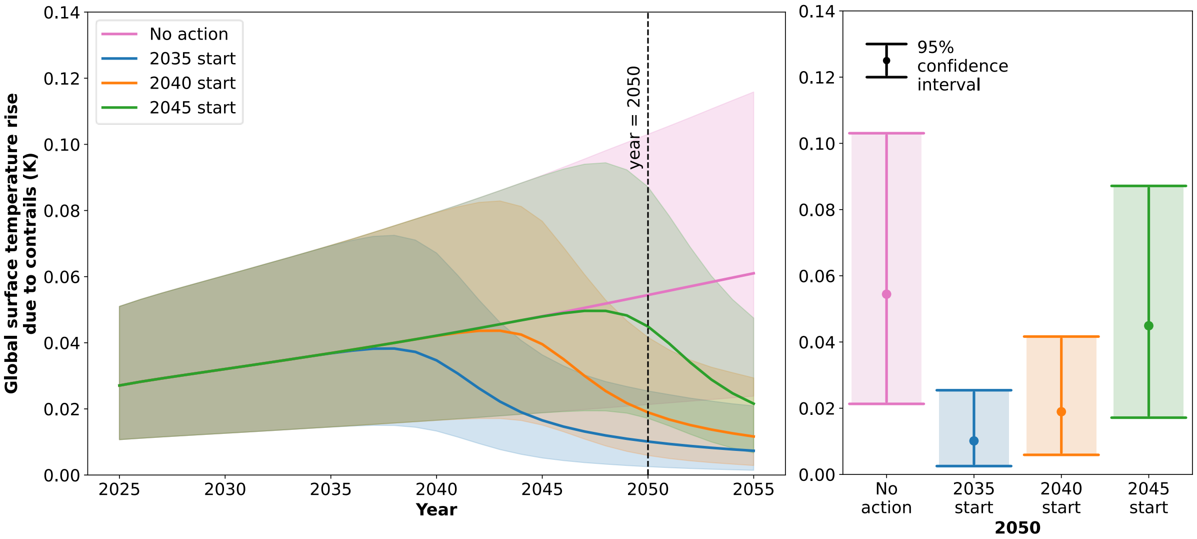

The distribution is presented from 2025 to 2055 (left) and for 2050 (right). The mean response (solid line) and 95% confidence intervals (shaded area) are depicted for contrail avoidance scenarios with start times of 2035, 2040, and 2045. All scenarios have 100% contrail avoidance effectiveness, and progress from no adoption to fleet wide adoption over the course of 10 years. The scenario where no contrail avoidance action is taken (No action) is also presented.

The aviation growth projection (AGP) AGP 0.21 is assumed, i.e. linear growth in flown distance beyond 2045 and a saturation in future contrail forcing effects.

Supplementary Figure 4 IV: The global surface temperature rise due to contrails, for contrail avoidance scenarios with different start dates.

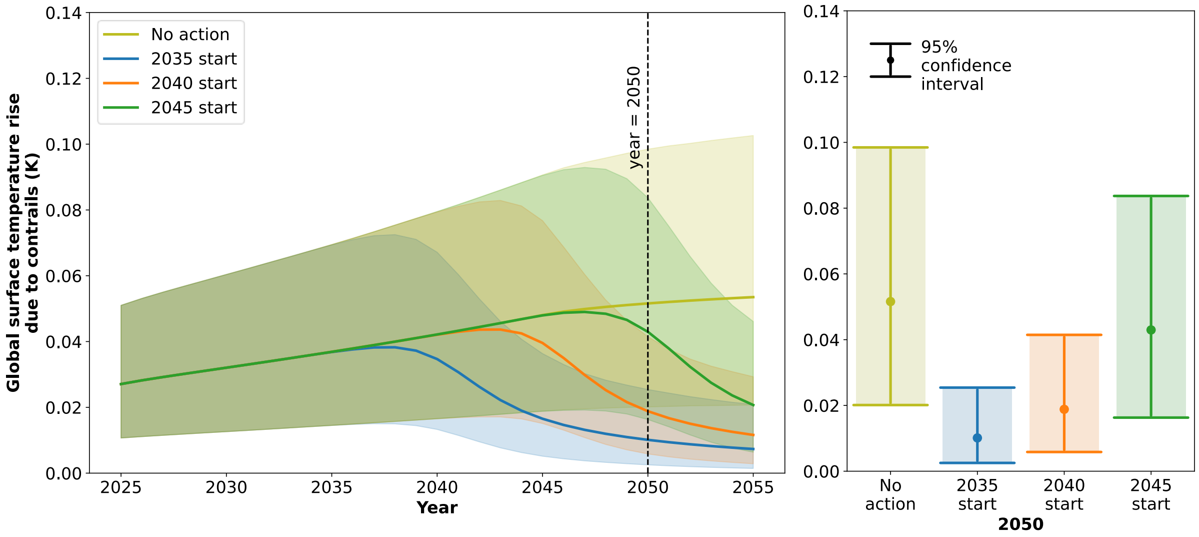

The distribution is presented from 2025 to 2055 (left) and for 2050 (right). The mean response (solid line) and 95% confidence intervals (shaded area) are depicted for contrail avoidance scenarios with start times of 2035, 2040, and 2045. All scenarios have 100% contrail avoidance effectiveness, and progress from no adoption to fleet wide adoption over the course of 10 years. The scenario where no contrail avoidance action is taken (No action) is also presented.

The aviation growth projection (AGP) AGP 0.14 is assumed, i.e. no growth in flown distance beyond 2045 and a saturation in future contrail forcing effects.

Supplementary Figure 5

Supplementary Figure 5 I: The global surface temperature rise due to contrails, for contrail avoidance scenarios with and without the modification of fleet wide fuel composition.

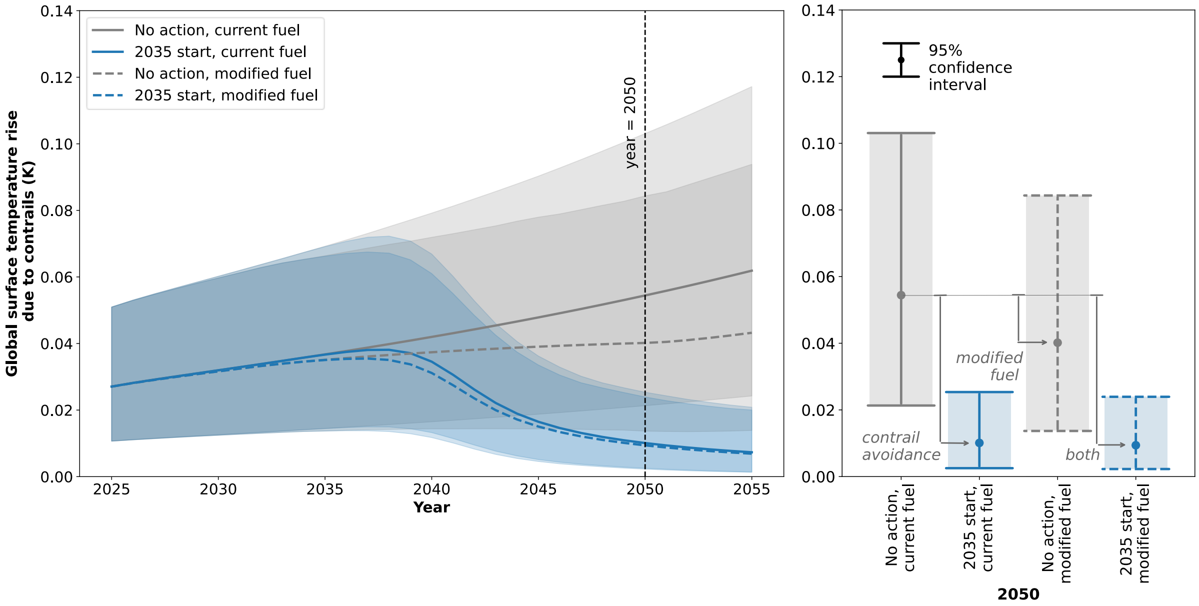

The distribution is presented from 2025 to 2055 (left) and for 2050 (right). The mean response with (dashed line) and without (solid line) modified fuel composition and 95% confidence intervals (shaded area) are depicted. All scenarios have a 2035 start date, have a 100% contrail avoidance effectiveness, and progress from no adoption to fleet wide adoption over the course of 10 years. The scenario where no contrail avoidance action is taken (No action) is also presented.

The aviation growth projection AGP (AGP) 0.29 is assumed, i.e. exponential growth in flown distance beyond 2045 and a saturation in future contrail forcing effects.

Supplementary Figure 5 II: The global surface temperature rise due to contrails, for contrail avoidance scenarios with and without the modification of fleet wide fuel composition.

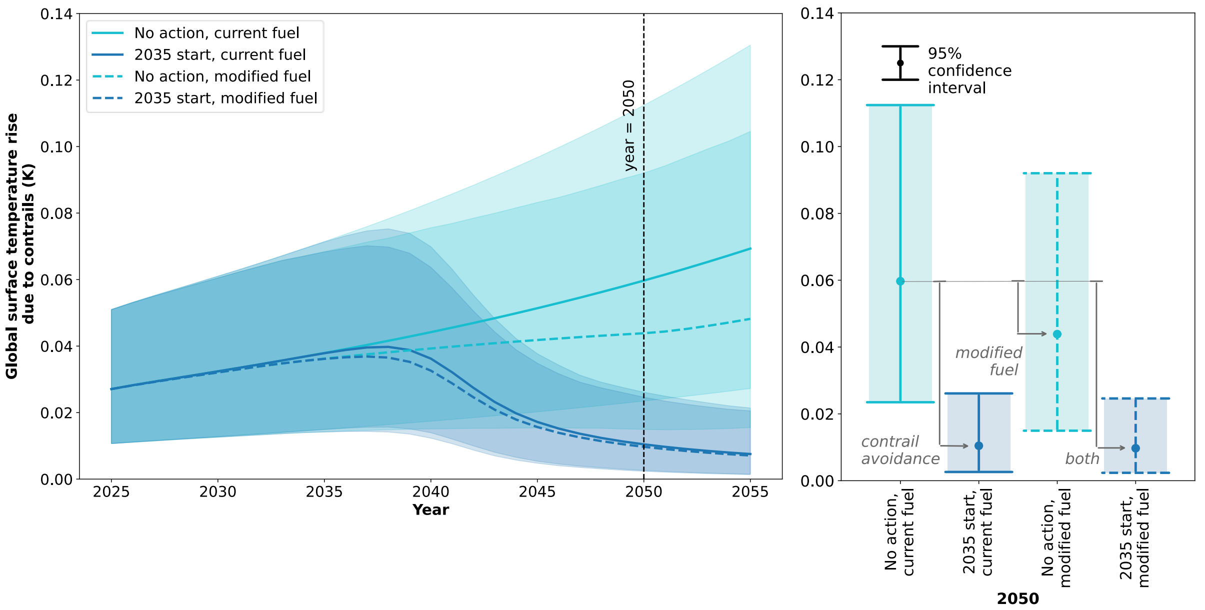

The distribution is presented from 2025 to 2055 (left) and for 2050 (right). The mean response with (dashed line) and without (solid line) modified fuel composition and 95% confidence intervals (shaded area) are depicted. All scenarios have a 2035 start date, have a 100% contrail avoidance effectiveness, and progress from no adoption to fleet wide adoption over the course of 10 years. The scenario where no contrail avoidance action is taken (No action) is also presented.

The aviation growth projection AGP (AGP) 0.36 is assumed, i.e. exponential growth in flown distance beyond 2045 and no saturation in future contrail forcing effects.

Supplementary Figure 5 III: The global surface temperature rise due to contrails, for contrail avoidance scenarios with and without the modification of fleet wide fuel composition.

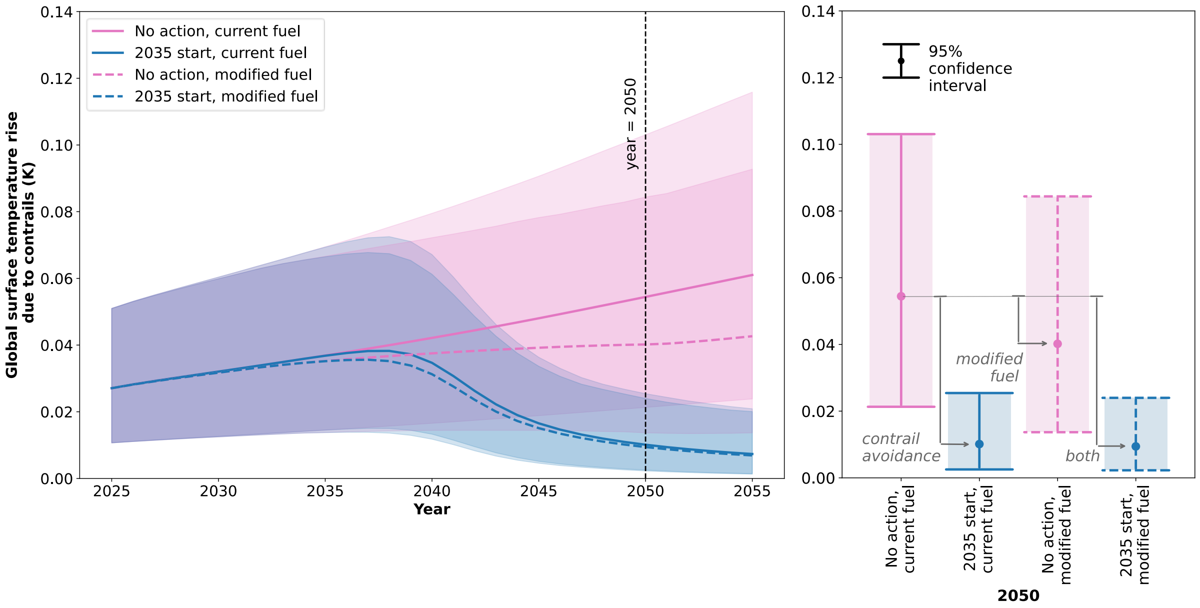

The distribution is presented from 2025 to 2055 (left) and for 2050 (right). The mean response with (dashed line) and without (solid line) modified fuel composition and 95% confidence intervals (shaded area) are depicted. All scenarios have a 2035 start date, have a 100% contrail avoidance effectiveness, and progress from no adoption to fleet wide adoption over the course of 10 years. The scenario where no contrail avoidance action is taken (No action) is also presented.

The aviation growth projection AGP (AGP) 0.21 is assumed, i.e. linear growth in flown distance beyond 2045 and a saturation in future contrail forcing effects.

Supplementary Figure 5 IV: The global surface temperature rise due to contrails, for contrail avoidance scenarios with and without the modification of fleet wide fuel composition.

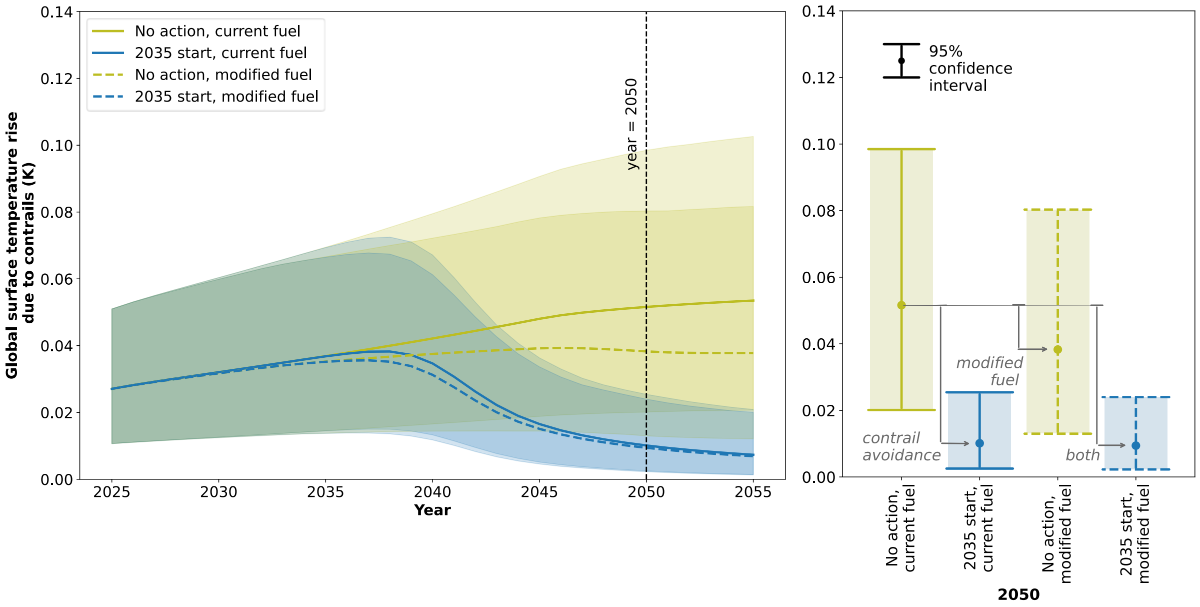

The distribution is presented from 2025 to 2055 (left) and for 2050 (right). The mean response with (dashed line) and without (solid line) modified fuel composition and 95% confidence intervals (shaded area) are depicted. All scenarios have a 2035 start date, have a 100% contrail avoidance effectiveness, and progress from no adoption to fleet wide adoption over the course of 10 years. The scenario where no contrail avoidance action is taken (No action) is also presented.

The aviation growth projection AGP (AGP) 0.14 is assumed, i.e. no growth in flown distance beyond 2045 and a saturation in future contrail forcing effects.

Supplementary Figure 6

Supplementary Figure 6 I: Distribution of the decrease in global surface temperature rise due to contrail avoidance and the temperature increase due to additional fuel burn in 2050 and 2100.

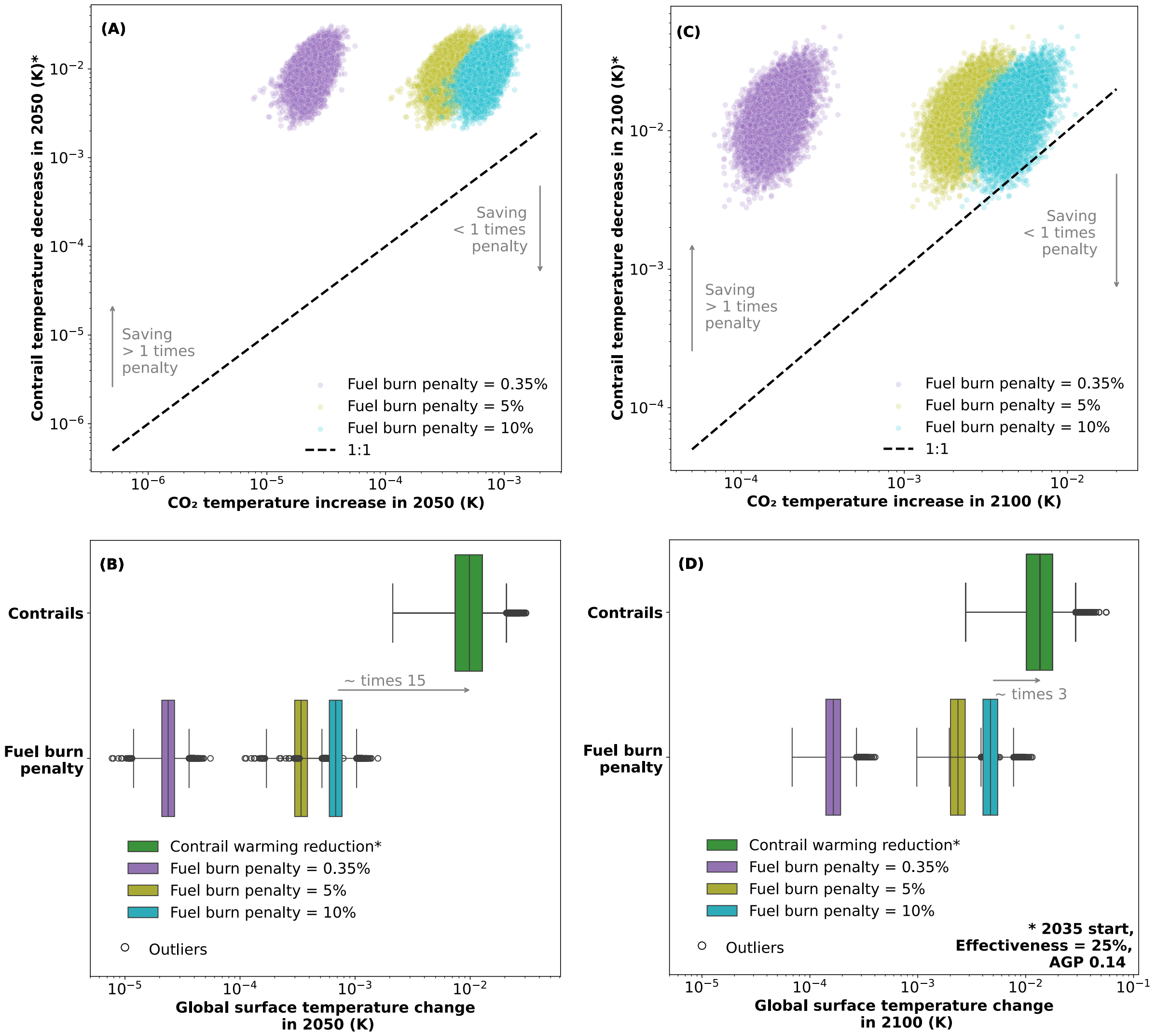

The distributions are shown for: 2050, (A) in a scatter plot and (B) in a box plot, and 2100, also (C) in a scatter plot and (D) in a box plot. Contrail avoidance is assumed 25% effective, the scenario start date is assumed to be 2035 and three levels of fuel burn penalty are presented: 0.35%, 5% and 10% of fleet averaged fuel burn at full adoption. Lines labelled “1:1” are shown in the scatter plots, which represent the level where the reduction in contrail warming due to contrail avoidance is equal to the CO_2 warming incurred due to the fuel burn penalty. The error bars, shaded areas, and central vertical line in the box plots indicate the 95% confidence intervals, the interquartile range, and the mean, respectively.

The aviation growth projection (AGP) AGP 0.14 is assumed, i.e. no growth in flown distance beyond 2045 and a saturation in future contrail forcing effects.

Supplementary Figure 6 II: Distribution of the decrease in global surface temperature rise due to contrail avoidance and the temperature increase due to additional fuel burn in 2050 and 2100.

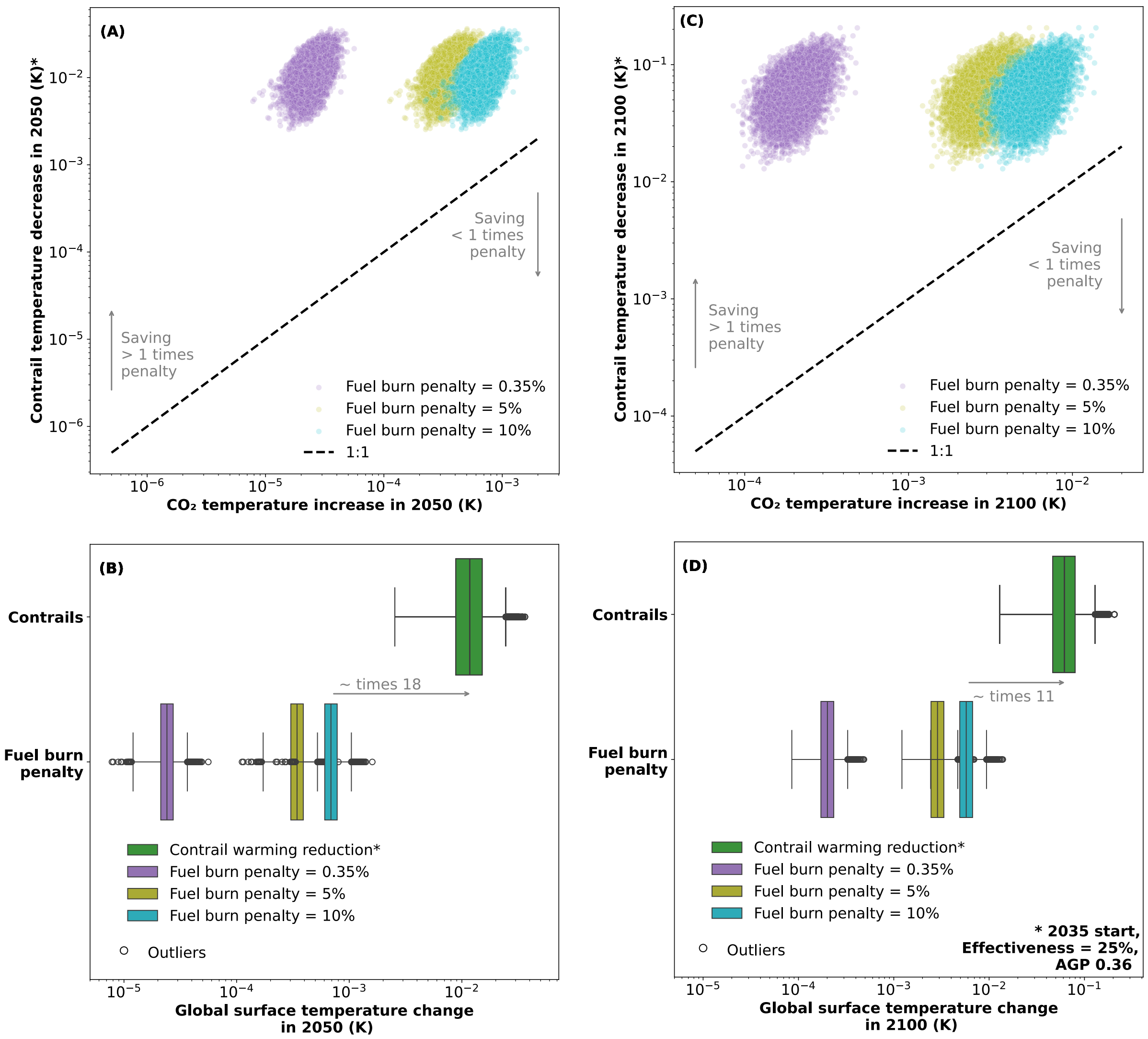

The distributions are shown for: 2050, (A) in a scatter plot and (B) in a box plot, and 2100, also (C) in a scatter plot and (D) in a box plot. Contrail avoidance is assumed 25% effective, the scenario start date is assumed to be 2035 and three levels of fuel burn penalty are presented: 0.35%, 5% and 10% of fleet averaged fuel burn at full adoption. Lines labelled “1:1” are shown in the scatter plots, which represent the level where the reduction in contrail warming due to contrail avoidance is equal to the CO_2 warming incurred due to the fuel burn penalty. The error bars, shaded areas, and central vertical line in the box plots indicate the 95% confidence intervals, the interquartile range, and the mean, respectively.

The aviation growth projection (AGP) AGP 0.36 is assumed, i.e. exponential growth in flown distance beyond 2045 and no saturation in future contrail forcing effects.

Supplementary Figure 6 III: Distribution of the decrease in global surface temperature rise due to contrail avoidance and the temperature increase due to additional fuel burn in 2050 and 2100.

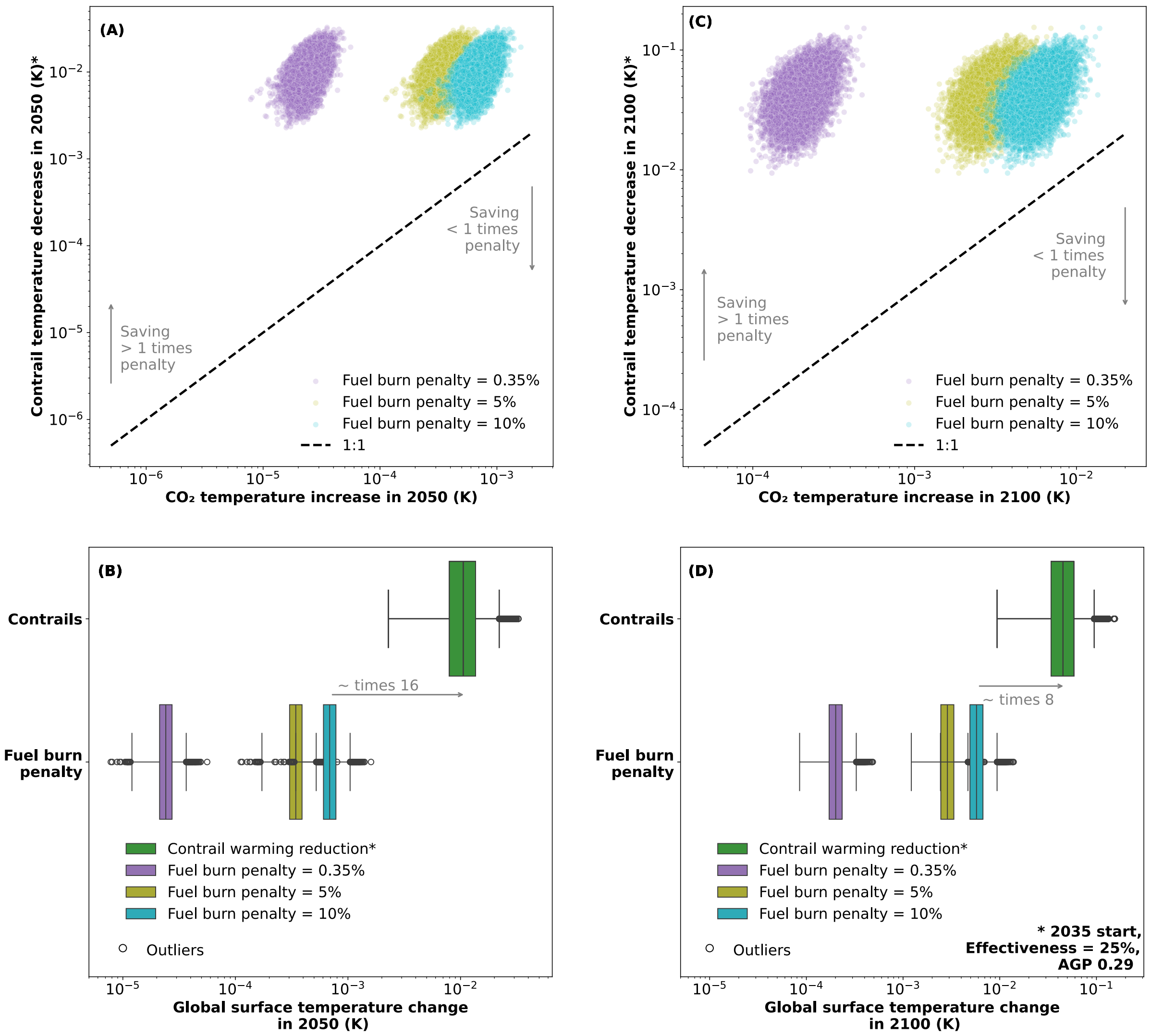

The distributions are shown for: 2050, (A) in a scatter plot and (B) in a box plot, and 2100, also (C) in a scatter plot and (D) in a box plot. Contrail avoidance is assumed 25% effective, the scenario start date is assumed to be 2035 and three levels of fuel burn penalty are presented: 0.35%, 5% and 10% of fleet averaged fuel burn at full adoption. Lines labelled “1:1” are shown in the scatter plots, which represent the level where the reduction in contrail warming due to contrail avoidance is equal to the CO_2 warming incurred due to the fuel burn penalty. The error bars, shaded areas, and central vertical line in the box plots indicate the 95% confidence intervals, the interquartile range, and the mean, respectively.

The aviation growth projection (AGP) AGP 0.29 is assumed, i.e. exponential growth in flown distance beyond 2045 and a saturation in future contrail forcing effects.

Supplementary Figure 6 IV: Distribution of the decrease in global surface temperature rise due to contrail avoidance and the temperature increase due to additional fuel burn in 2050 and 2100.

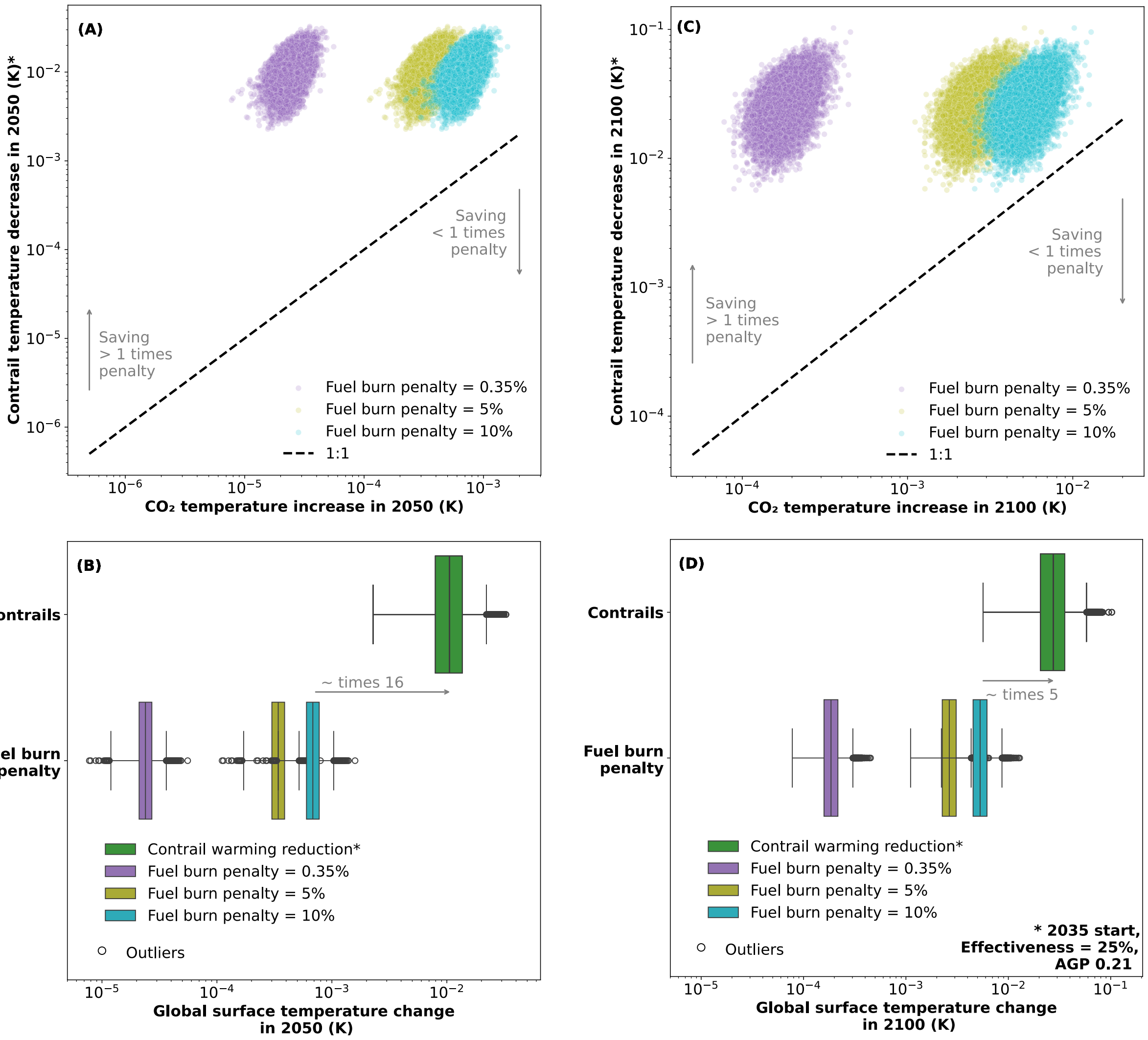

The distributions are shown for: 2050, (A) in a scatter plot and (B) in a box plot, and 2100, also (C) in a scatter plot and (D) in a box plot. Contrail avoidance is assumed 25% effective, the scenario start date is assumed to be 2035 and three levels of fuel burn penalty are presented: 0.35%, 5% and 10% of fleet averaged fuel burn at full adoption. Lines labelled “1:1” are shown in the scatter plots, which represent the level where the reduction in contrail warming due to contrail avoidance is equal to the CO_2 warming incurred due to the fuel burn penalty. The error bars, shaded areas, and central vertical line in the box plots indicate the 95% confidence intervals, the interquartile range, and the mean, respectively.

The aviation growth projection (AGP) AGP 0.21 is assumed, i.e. linear growth in flown distance beyond 2045 and a saturation in future contrail forcing effects.

Supplementary Figure 7

Supplementary Figure 7 I: A summary of global surface temperature change in 2050, due to contrail avoidance and its associated fuel burn penalty.

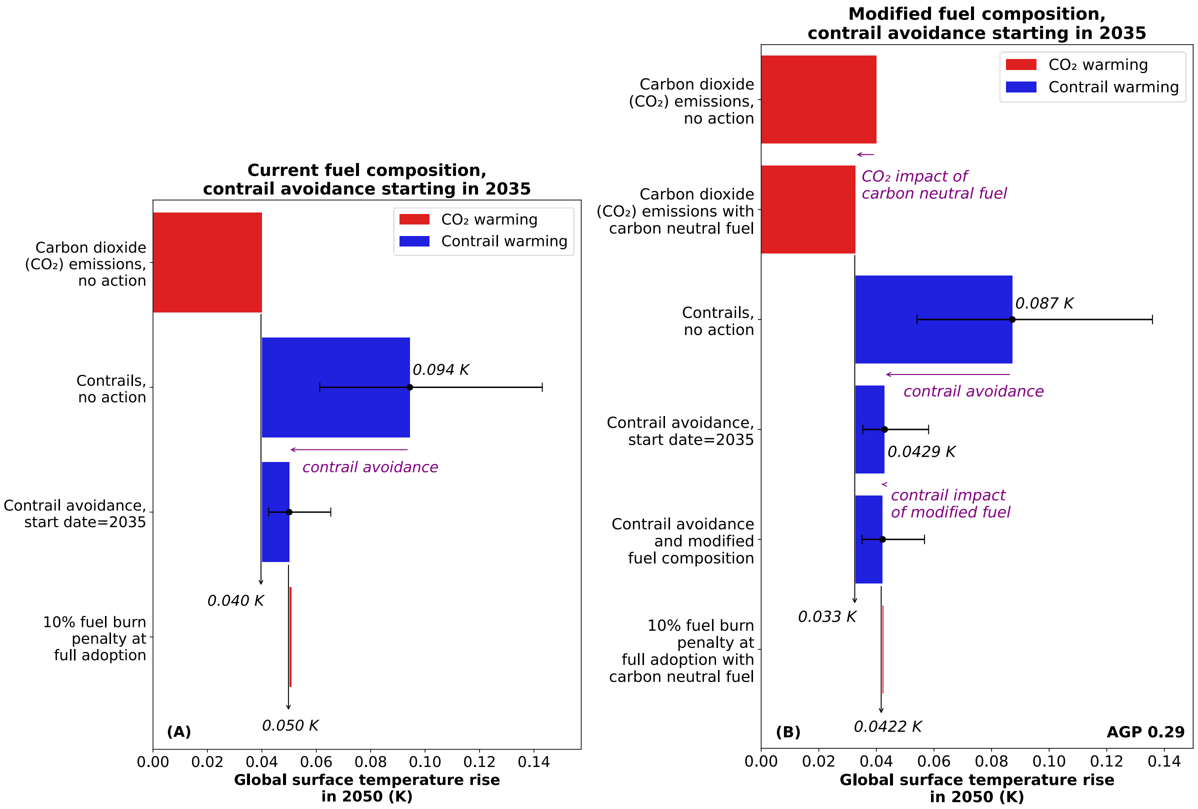

The scenario is presented (A) without and (B) with modifications to fuel composition. A start date of 2035, a 10-year period between start date and full adoption, a 100% contrail avoidance effectiveness, and a 10% fuel burn penalty at full adoption are assumed. The temperature impact of all aviation carbon dioxide emissions is reduced (but not eliminated) by the introduction of carbon neutral fuel, which is assumed to be phased in alongside the modifications to fuel composition, starting in 2035. All error bars indicate 95% confidence intervals.

The aviation growth projection (AGP) AGP 0.29 is assumed, i.e. exponential growth in flown distance beyond 2045 and a saturation in future contrail forcing effects.

Supplementary Figure 7 II: A summary of global surface temperature change in 2050, due to contrail avoidance and its associated fuel burn penalty.

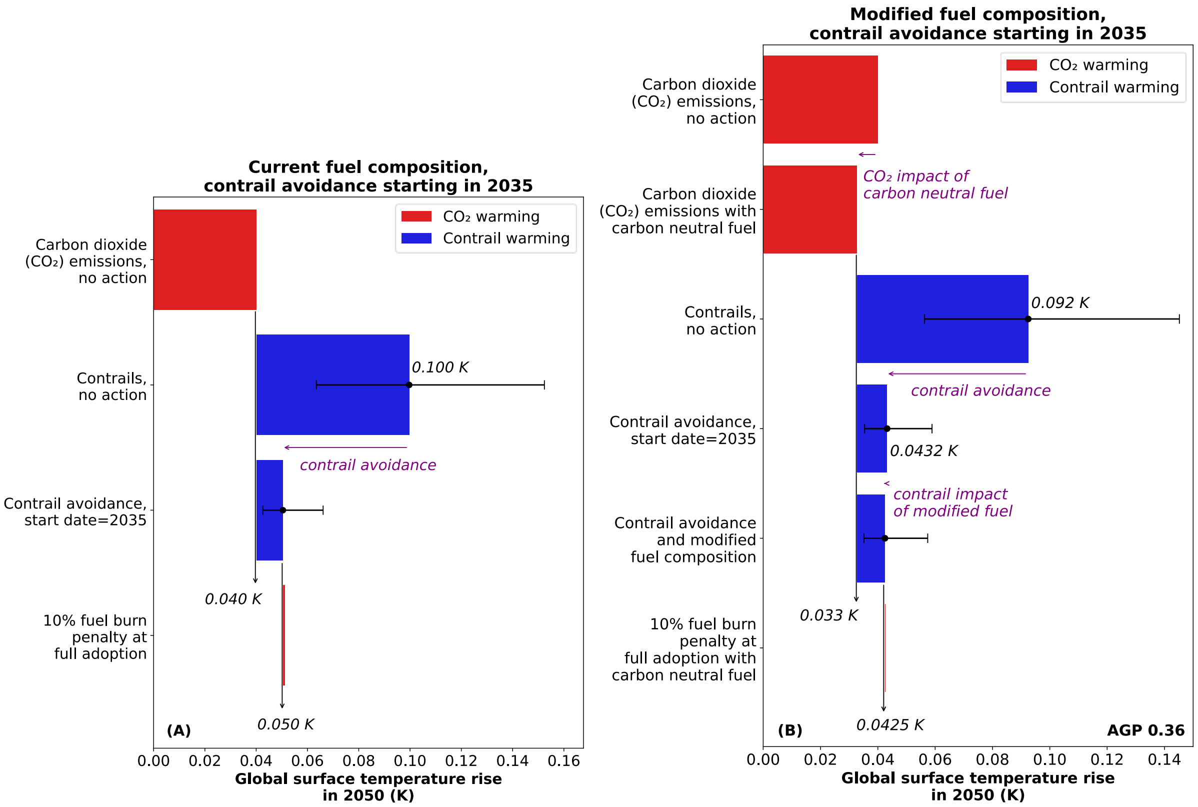

The scenario is presented (A) without and (B) with modifications to fuel composition. A start date of 2035, a 10-year period between start date and full adoption, a 100% contrail avoidance effectiveness, and a 10% fuel burn penalty at full adoption are assumed. The temperature impact of all aviation carbon dioxide emissions is reduced (but not eliminated) by the introduction of carbon neutral fuel, which is assumed to be phased in alongside the modifications to fuel composition, starting in 2035. All error bars indicate 95% confidence intervals.

The aviation growth projection (AGP) AGP 0.36 is assumed, i.e. exponential growth in flown distance beyond 2045 and no saturation in future contrail forcing effects.

Supplementary Figure 7 III: A summary of global surface temperature change in 2050, due to contrail avoidance and its associated fuel burn penalty.

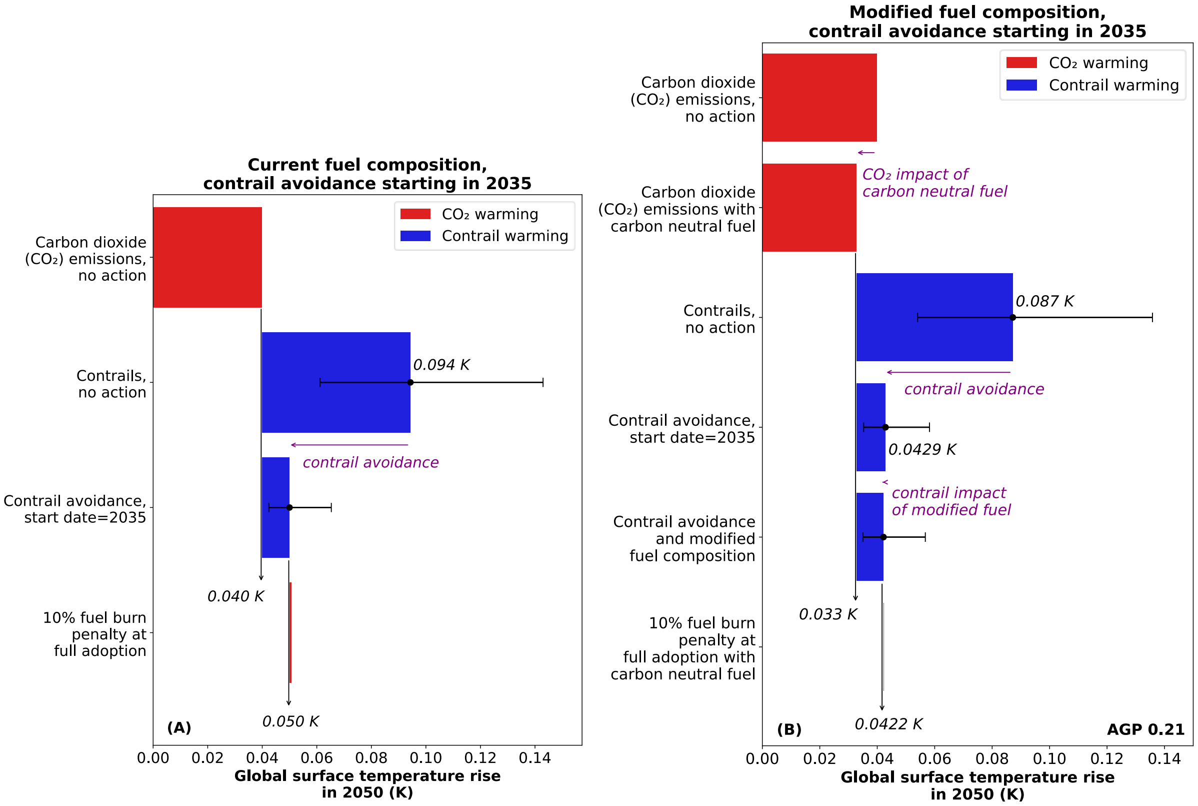

The scenario is presented (A) without and (B) with modifications to fuel composition. A start date of 2035, a 10-year period between start date and full adoption, a 100% contrail avoidance effectiveness, and a 10% fuel burn penalty at full adoption are assumed. The temperature impact of all aviation carbon dioxide emissions is reduced (but not eliminated) by the introduction of carbon neutral fuel, which is assumed to be phased in alongside the modifications to fuel composition, starting in 2035. All error bars indicate 95% confidence intervals.

The aviation growth projection AGP 0.21 is assumed, i.e. linear growth in flown distance beyond 2045 and a saturation in future contrail forcing effects.

Supplementary Figure 7 IV: A summary of global surface temperature change in 2050, due to contrail avoidance and its associated fuel burn penalty.

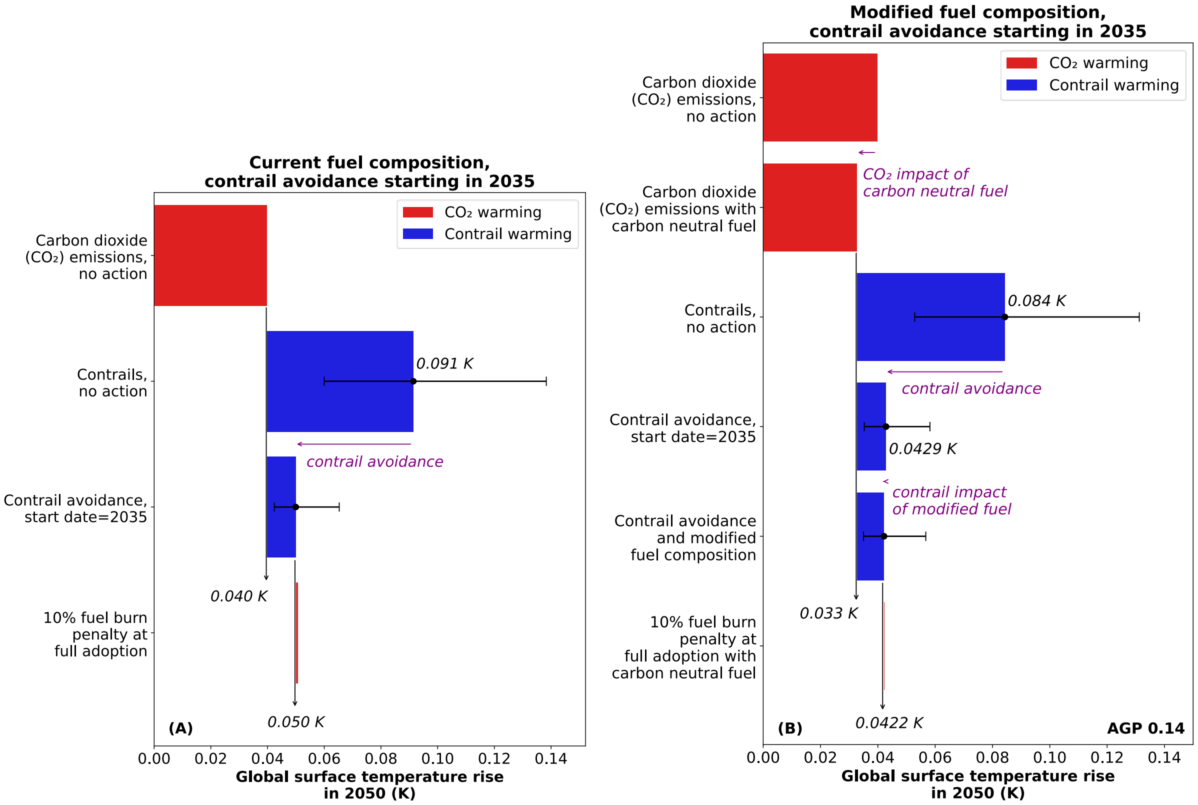

The scenario is presented (A) without and (B) with modifications to fuel composition. A start date of 2035, a 10-year period between start date and full adoption, a 100% contrail avoidance effectiveness, and a 10% fuel burn penalty at full adoption are assumed. The temperature impact of all aviation carbon dioxide emissions is reduced (but not eliminated) by the introduction of carbon neutral fuel, which is assumed to be phased in alongside the modifications to fuel composition, starting in 2035. All error bars indicate 95% confidence intervals.

The aviation growth projection AGP 0.14 is assumed, i.e. no growth in flown distance beyond 2045 and a saturation in future contrail forcing effects.
